# Hexa-μ_2_-acetato-triaqua-μ_3_-oxido-triiron(III) nitrate acetic acid solvate

**DOI:** 10.1107/S1600536808019806

**Published:** 2008-07-05

**Authors:** Sumei Yao, Jianhua Liu, Qiuxia Han

**Affiliations:** a Medical College of Henan University, Henan University, Kaifeng 475004, People’s Republic of China; b Basic Experiment Teaching Center, Henan University, Kaifeng 475004, People’s Republic of China

## Abstract

The asymmetric unit of the title compound, [Fe_3_(CH_3_COO)_6_O(H_2_O)_3_]NO_3_·CH_3_COOH, consists of a hexa-μ_2_-acetato-triaqua-μ_3_-oxo-triiron(III) macrocation, a nitrate ion and an acetic acid solvent mol­ecule. In the cation, each Fe^3+^ ion is coordinated by four carboxyl­ate O atoms, one central bridged O atom and one water mol­ecule, resulting in distorted FeO_6_ octa­hedra. A network of O—H⋯O hydrogen bonds helps to establish the packing.

## Related literature

For related literature, see: Fujihara *et al.* (1998 ▶); Ren *et al.* (2004 ▶); Thirumurugan & Natarajan (2004 ▶); Vrubel *et al.* (2006 ▶); Zhang *et al.* (2005 ▶).
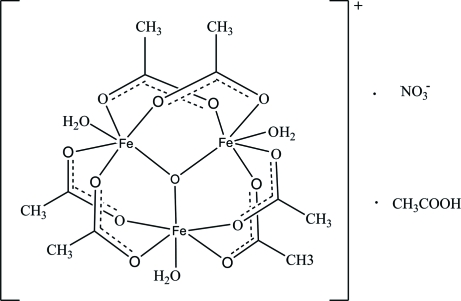


## Experimental

### 

#### Crystal data

[Fe_3_(C_2_H_3_O_2_)_6_O(H_2_O)_3_]NO_3_·C_2_H_4_O_2_
*M* *_r_* = 713.92
Monoclinic, 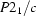

*a* = 11.835 (3) Å
*b* = 14.755 (4) Å
*c* = 15.250 (4) Å
β = 90.851 (5)°
*V* = 2662.8 (12) Å^3^
*Z* = 4
Mo *K*α radiation
μ = 1.71 mm^−1^
*T* = 296 (2) K
0.18 × 0.13 × 0.10 mm

#### Data collection

Bruker SMART CCD diffractometer
Absorption correction: multi-scan (*SADABS*; Sheldrick, 2001 ▶) *T*_min_ = 0.750, *T*_max_ = 0.848
14072 measured reflections
4953 independent reflections
3355 reflections with *I* > 2σ(*I*)
*R*_int_ = 0.054

#### Refinement

*R*[*F* ^2^ > 2σ(*F* ^2^)] = 0.044
*wR*(*F* ^2^) = 0.106
*S* = 1.00
4953 reflections
378 parameters
9 restraints
H atoms treated by a mixture of independent and constrained refinement
Δρ_max_ = 0.51 e Å^−3^
Δρ_min_ = −0.41 e Å^−3^

### 

Data collection: *SMART* (Bruker, 2001 ▶); cell refinement and data reduction: *SAINT-Plus* (Bruker, 2001 ▶); structure solution: *SHELXS97* (Sheldrick, 2008 ▶); structure refinement: *SHELXL97* (Sheldrick, 2008 ▶); molecular graphics: *PLATON* (Spek, 2003 ▶); software used to prepare material for publication: *PLATON*.

## Supplementary Material

Crystal structure: contains datablocks global, I. DOI: 10.1107/S1600536808019806/hb2740sup1.cif

Structure factors: contains datablocks I. DOI: 10.1107/S1600536808019806/hb2740Isup2.hkl

Additional supplementary materials:  crystallographic information; 3D view; checkCIF report

## Figures and Tables

**Table 1:** Selected bond lengths (Å)

Fe1—O13	1.897 (2)
Fe1—O1	1.987 (2)
Fe1—O10	1.995 (2)
Fe1—O12	2.005 (3)
Fe1—O3	2.063 (2)
Fe1—O1*W*	2.104 (2)
Fe2—O13	1.900 (2)
Fe2—O5	1.985 (2)
Fe2—O7	2.021 (2)
Fe2—O2	2.030 (2)
Fe2—O4	2.030 (2)
Fe2—O2*W*	2.126 (3)
Fe3—O13	1.916 (2)
Fe3—O11	2.011 (3)
Fe3—O6	2.013 (2)
Fe3—O8	2.013 (2)
Fe3—O9	2.017 (2)
Fe3—O3*W*	2.048 (2)

**Table 2:** Hydrogen-bond geometry (Å, °)

*D*—H⋯*A*	*D*—H	H⋯*A*	*D*⋯*A*	*D*—H⋯*A*
O14—H14⋯O17^i^	0.82	1.82	2.642 (4)	178
O3*W*—H3*AW*⋯O15^ii^	0.816 (9)	1.894 (9)	2.697 (4)	168 (2)
O1*W*—H1*AW*⋯O18^iii^	0.815 (9)	2.008 (10)	2.821 (4)	176 (2)
O3*W*—H3*BW*⋯O17^iv^	0.818 (9)	1.938 (13)	2.742 (4)	167 (3)
O2*W*—H2*AW*⋯O15^v^	0.816 (9)	2.28 (2)	2.904 (4)	134 (2)
O1*W*—H1*BW*⋯O3^vi^	0.814 (9)	2.188 (12)	2.948 (3)	155 (2)

Symmetry codes: (i) 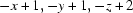; (ii) 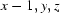; (iii) 
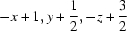
; (iv) 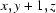; (v) 
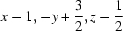
; (vi) 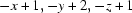.

**Fig. 1.**
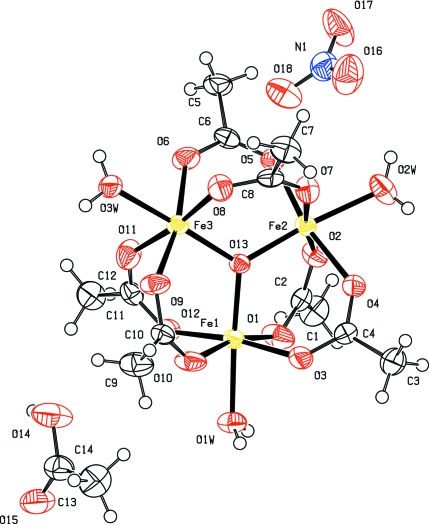
The molecular structure of (I), with displacement ellipsoids for the non-hydrogen atoms drawn at the 50% probability level.

**Fig. 2.**
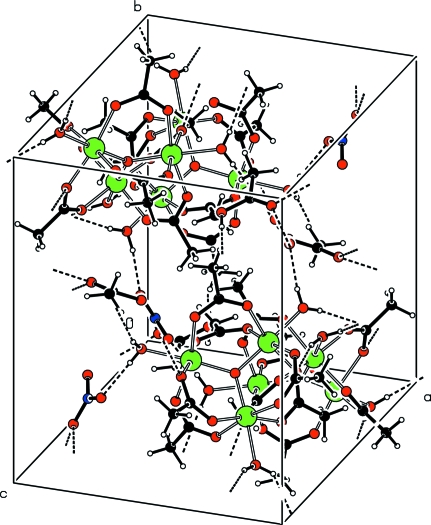
Three-dimensional structure of (I), with hydrogen bonds shown as dashed lines.

## supplementary crystallographic information

### Comment

Transiton-metal coordination complexes based on carboxylates have been attracting chemist's interests and constitutes one of the widest families of research (Thirumurugan & Natarajan, 2004). During the past years, lots of novel carboxylates compounds have been reported (Zhang *et al.*, 2005), in which carboxlate-supported Cr_3_(/m_3_-O) (Fujihara *et al.*, 1998) and Fe_3_(/m_3_-O) core (Ren *et al.*, 2004; Vrubel *et al.*, 2006), present two large kinds of widely investigated transtion-metal complexes. Herein, we report the title compound (I).

The title compound, (I), presents a macrocation of [Fe_3_O(CH_3_COO)_6_(H_2_O)_3_]^+^, in which Fe^3+^ is coordinated by four oxygen atoms from four carboxylates of four acetate anions, one central bridged oxygen atom, and one water molecule. The environment of all the Fe ions are distorted octahedral geometry (Fig. 1). The three Fe atoms approximatively reside in an equilateral triangle with an oxide ion in the center [Fe_3_O]. The Fe—O distances range from 1.897 (2) to 2.126 (3) Å (Table 1).

In the crystal, the components are linked by O—H···O hydrogen bonds generating a three-dimensional framework (Fig. 2 and Table 2).

### Experimental

Fe(NO_3_)_3_.9H_2_O (1 mmol, 0.404 g) was suspended in 5 ml water and 3 ml (1 mol/*L*) NaOH solution was added dropwise to produce a brown precipitate, then 25 ml acetic acid were added to the mixture. It was stirred under reflux for 3 h. The solution was filtered, and the filtrate was kept at the room temperature. After one weeks, xxx blocks of (I) were obtained.

### Refinement

H atoms were treated as riding, with C—H distances in the range of 0.93–0.98 Å and O—H distances of 0.82 Å, and were refined as riding with *U*_iso_(*H*)=1.2*U*_eq_(C_methylene_ and C_methylidyne_) and *U*_iso_(H)=1.5*U*_eq_(O or C_methyl_).

### Crystal data

**Table:**

[Fe_3_(C_2_H_3_O_2_)_6_O(H_2_O)_3_]NO_3_·C_2_H_4_O_2_	*F*_000_ = 1460
*M**_r_* = 713.92	*D*_x_ = 1.781 Mg m^−^^3^
Monoclinic, *P*2_1_/*c*	Mo *K*α radiation λ = 0.71073 Å
Hall symbol: -P 2ybc	Cell parameters from 2092 reflections
*a* = 11.835 (3) Å	θ = 2.2–23.2º
*b* = 14.755 (4) Å	µ = 1.71 mm^−^^1^
*c* = 15.250 (4) Å	*T* = 296 (2) K
β = 90.851 (5)º	Block, yellow
*V* = 2662.8 (12) Å^3^	0.18 × 0.13 × 0.10 mm
*Z* = 4	

### Data collection

**Table:**

Bruker SMART CCD diffractometer	4953 independent reflections
Radiation source: fine-focus sealed tube	3355 reflections with *I* > 2σ(*I*)
Monochromator: graphite	*R*_int_ = 0.054
*T* = 296(2) K	θ_max_ = 25.5º
ω scans	θ_min_ = 1.7º
Absorption correction: multi-scan(SADABS; Sheldrick, 2001)	*h* = −14→11
*T*_min_ = 0.750, *T*_max_ = 0.848	*k* = −17→17
14072 measured reflections	*l* = −18→17

### Refinement

**Table:**

Refinement on *F*^2^	Secondary atom site location: difference Fourier map
Least-squares matrix: full	Hydrogen site location: inferred from neighbouring sites
*R*[*F*^2^ > 2σ(*F*^2^)] = 0.044	H atoms treated by a mixture of independent and constrained refinement
*wR*(*F*^2^) = 0.106	*w* = 1/[σ^2^(*F*_o_^2^) + (0.048*P*)^2^] where *P* = (*F*_o_^2^ + 2*F*_c_^2^)/3
*S* = 1.00	(Δ/σ)_max_ = 0.001
4953 reflections	Δρ_max_ = 0.51 e Å^−^^3^
378 parameters	Δρ_min_ = −0.41 e Å^−^^3^
9 restraints	Extinction correction: none
Primary atom site location: structure-invariant direct methods	

### Special details

**Table:**

Geometry. All e.s.d.'s (except the e.s.d. in the dihedral angle between two l.s. planes) are estimated using the full covariance matrix. The cell e.s.d.'s are taken into account individually in the estimation of e.s.d.'s in distances, angles and torsion angles; correlations between e.s.d.'s in cell parameters are only used when they are defined by crystal symmetry. An approximate (isotropic) treatment of cell e.s.d.'s is used for estimating e.s.d.'s involving l.s. planes.
Refinement. Refinement of *F*^2^ against ALL reflections. The weighted *R*-factor *wR* and goodness of fit *S* are based on *F*^2^, conventional *R*-factors *R* are based on *F*, with *F* set to zero for negative *F*^2^. The threshold expression of *F*^2^ > σ(*F*^2^) is used only for calculating *R*-factors(gt) *etc*. and is not relevant to the choice of reflections for refinement. *R*-factors based on *F*^2^ are statistically about twice as large as those based on *F*, and *R*- factors based on ALL data will be even larger.

### Fractional atomic coordinates and isotropic or equivalent isotropic displacement parameters (Å^2^)

**Table:**

	*x*	*y*	*z*	*U*_iso_*/*U*_eq_	
Fe1	0.40305 (4)	0.89656 (3)	0.62790 (3)	0.02692 (12)	
Fe2	0.17864 (4)	0.76827 (3)	0.59152 (3)	0.02751 (12)	
Fe3	0.16879 (4)	0.93399 (3)	0.73634 (3)	0.02779 (13)	
O1W	0.5730 (2)	0.92274 (16)	0.59831 (17)	0.0390 (7)	
O2W	0.1067 (2)	0.66185 (17)	0.51505 (18)	0.0536 (8)	
O3W	0.0853 (2)	1.01043 (16)	0.82684 (16)	0.0414 (7)	
O1	0.4549 (2)	0.76906 (16)	0.61642 (19)	0.0505 (8)	
O2	0.30533 (19)	0.67883 (15)	0.61664 (16)	0.0373 (6)	
O3	0.3729 (2)	0.90801 (17)	0.49476 (15)	0.0433 (7)	
O4	0.2469 (2)	0.79746 (15)	0.47350 (15)	0.0375 (6)	
O5	0.0995 (2)	0.71685 (15)	0.69403 (15)	0.0379 (6)	
O6	0.0942 (2)	0.82725 (15)	0.79378 (16)	0.0461 (7)	
O7	0.0395 (2)	0.84025 (16)	0.55730 (15)	0.0387 (7)	
O8	0.03183 (19)	0.94965 (16)	0.65798 (16)	0.0406 (7)	
O9	0.2290 (2)	1.05548 (15)	0.69826 (17)	0.0430 (7)	
O10	0.38902 (19)	1.03126 (16)	0.62901 (17)	0.0456 (7)	
O11	0.2928 (2)	0.92041 (18)	0.82695 (16)	0.0492 (8)	
O12	0.4514 (2)	0.89345 (18)	0.75438 (16)	0.0481 (7)	
O13	0.25047 (17)	0.86634 (13)	0.65078 (14)	0.0254 (5)	
O17	0.1754 (3)	0.16154 (19)	0.9058 (2)	0.0782 (11)	
O18	0.2920 (3)	0.26869 (19)	0.9325 (2)	0.0674 (9)	
O14	0.7459 (2)	0.9001 (2)	0.9425 (2)	0.0738 (10)	
H14	0.7698	0.8798	0.9893	0.111*	
O15	0.9248 (2)	0.9381 (2)	0.92925 (19)	0.0637 (9)	
C1	0.4856 (3)	0.6134 (2)	0.6411 (3)	0.0472 (11)	
H1A	0.4414	0.5590	0.6445	0.071*	
H1B	0.5258	0.6224	0.6956	0.071*	
H1C	0.5386	0.6080	0.5943	0.071*	
C2	0.4094 (3)	0.6923 (2)	0.6240 (2)	0.0302 (8)	
C3	0.3290 (3)	0.8632 (3)	0.3488 (2)	0.0479 (11)	
H3A	0.3837	0.9093	0.3361	0.072*	
H3B	0.2576	0.8792	0.3225	0.072*	
H3C	0.3539	0.8063	0.3256	0.072*	
C4	0.3164 (3)	0.8553 (2)	0.4464 (2)	0.0305 (9)	
C5	0.0056 (3)	0.6886 (3)	0.8275 (2)	0.0458 (11)	
H5A	−0.0075	0.6307	0.8004	0.069*	
H5B	−0.0655	0.7167	0.8400	0.069*	
H5C	0.0478	0.6803	0.8811	0.069*	
C6	0.0711 (3)	0.7479 (2)	0.7666 (2)	0.0317 (9)	
C7	−0.1126 (3)	0.9443 (3)	0.5496 (3)	0.0476 (11)	
H7A	−0.1380	0.9960	0.5820	0.071*	
H7B	−0.1696	0.8980	0.5504	0.071*	
H7C	−0.0987	0.9618	0.4901	0.071*	
C8	−0.0058 (3)	0.9087 (2)	0.5905 (2)	0.0317 (9)	
C9	0.3287 (3)	1.1823 (2)	0.6455 (3)	0.0512 (12)	
H9A	0.3975	1.1935	0.6147	0.077*	
H9B	0.3322	1.2118	0.7016	0.077*	
H9C	0.2659	1.2056	0.6120	0.077*	
C10	0.3142 (3)	1.0824 (2)	0.6584 (2)	0.0303 (9)	
C11	0.4539 (3)	0.8788 (3)	0.9092 (2)	0.0487 (11)	
H11A	0.4025	0.8865	0.9567	0.073*	
H11B	0.5168	0.9193	0.9168	0.073*	
H11C	0.4807	0.8174	0.9084	0.073*	
C12	0.3942 (3)	0.8995 (2)	0.8241 (2)	0.0324 (9)	
O16	0.1840 (3)	0.2745 (2)	0.8165 (2)	0.0802 (11)	
N1	0.2187 (3)	0.2358 (2)	0.8830 (2)	0.0534 (10)	
C13	0.7984 (4)	0.9702 (3)	0.8110 (3)	0.0624 (13)	
H13A	0.7694	1.0306	0.8163	0.094*	
H13B	0.7415	0.9318	0.7851	0.094*	
H13C	0.8637	0.9707	0.7744	0.094*	
C14	0.8301 (3)	0.9353 (3)	0.8986 (3)	0.0470 (11)	
H3AW	0.0417 (12)	0.9921 (13)	0.8637 (11)	0.083 (16)*	
H1AW	0.6123 (14)	0.8779 (8)	0.5921 (16)	0.048 (12)*	
H3BW	0.1149 (16)	1.0579 (9)	0.8427 (17)	0.085 (16)*	
H2AW	0.0434 (9)	0.6407 (19)	0.5202 (14)	0.106 (19)*	
H2BW	0.122 (2)	0.667 (3)	0.4630 (7)	0.16 (3)*	
H1BW	0.584 (3)	0.9594 (9)	0.5594 (11)	0.079 (16)*	

### Atomic displacement parameters (Å^2^)

**Table:**

	*U*^11^	*U*^22^	*U*^33^	*U*^12^	*U*^13^	*U*^23^
Fe1	0.0246 (3)	0.0245 (2)	0.0317 (3)	−0.0017 (2)	0.0026 (2)	−0.0008 (2)
Fe2	0.0289 (3)	0.0224 (2)	0.0313 (3)	−0.0028 (2)	0.0011 (2)	0.0004 (2)
Fe3	0.0276 (3)	0.0232 (2)	0.0327 (3)	−0.0019 (2)	0.0055 (2)	−0.0002 (2)
O1W	0.0293 (13)	0.0336 (13)	0.0542 (16)	0.0006 (11)	0.0079 (12)	0.0048 (12)
O2W	0.0598 (18)	0.0415 (16)	0.0590 (19)	−0.0159 (14)	−0.0129 (15)	−0.0030 (14)
O3W	0.0436 (15)	0.0338 (14)	0.0472 (15)	−0.0071 (12)	0.0163 (13)	−0.0110 (12)
O1	0.0332 (14)	0.0288 (13)	0.090 (2)	0.0000 (12)	0.0072 (14)	−0.0050 (14)
O2	0.0375 (14)	0.0235 (12)	0.0506 (15)	0.0018 (11)	−0.0025 (12)	−0.0014 (11)
O3	0.0430 (15)	0.0563 (16)	0.0306 (13)	−0.0180 (13)	0.0012 (12)	0.0004 (12)
O4	0.0425 (14)	0.0368 (13)	0.0333 (13)	−0.0113 (12)	0.0046 (11)	−0.0004 (11)
O5	0.0445 (15)	0.0337 (13)	0.0356 (14)	−0.0096 (12)	0.0077 (12)	0.0040 (11)
O6	0.0565 (16)	0.0293 (14)	0.0530 (16)	−0.0106 (12)	0.0223 (13)	0.0000 (12)
O7	0.0382 (14)	0.0376 (14)	0.0399 (14)	0.0067 (12)	−0.0069 (12)	−0.0032 (12)
O8	0.0323 (14)	0.0413 (14)	0.0482 (16)	0.0094 (12)	−0.0028 (12)	−0.0082 (12)
O9	0.0410 (15)	0.0247 (13)	0.0638 (17)	−0.0030 (11)	0.0204 (13)	0.0019 (12)
O10	0.0341 (14)	0.0250 (13)	0.0784 (19)	−0.0050 (11)	0.0198 (14)	−0.0003 (13)
O11	0.0411 (15)	0.0685 (18)	0.0376 (15)	0.0119 (14)	−0.0061 (13)	−0.0085 (13)
O12	0.0355 (14)	0.0763 (19)	0.0324 (14)	−0.0086 (14)	−0.0018 (12)	0.0012 (14)
O13	0.0236 (12)	0.0226 (11)	0.0303 (12)	−0.0007 (9)	0.0018 (10)	−0.0004 (10)
O17	0.116 (3)	0.0492 (18)	0.069 (2)	−0.0405 (18)	−0.019 (2)	0.0065 (16)
O18	0.0604 (19)	0.0547 (18)	0.087 (2)	−0.0155 (16)	−0.0065 (18)	0.0070 (17)
O14	0.0485 (18)	0.096 (2)	0.076 (2)	−0.0236 (18)	−0.0037 (16)	0.0325 (19)
O15	0.0425 (17)	0.089 (2)	0.0599 (19)	−0.0098 (16)	0.0070 (15)	0.0113 (17)
C1	0.050 (2)	0.038 (2)	0.053 (2)	0.0169 (19)	0.000 (2)	0.0056 (19)
C2	0.034 (2)	0.0310 (18)	0.0254 (18)	0.0068 (17)	0.0036 (15)	−0.0009 (15)
C3	0.053 (2)	0.060 (3)	0.031 (2)	−0.006 (2)	0.0001 (19)	0.0040 (19)
C4	0.0294 (19)	0.0318 (19)	0.0305 (19)	0.0023 (16)	0.0027 (16)	0.0025 (16)
C5	0.047 (2)	0.047 (2)	0.043 (2)	−0.017 (2)	0.0054 (19)	0.0111 (19)
C6	0.0297 (19)	0.0257 (19)	0.040 (2)	−0.0044 (15)	−0.0030 (16)	0.0085 (15)
C7	0.039 (2)	0.049 (2)	0.054 (3)	0.0014 (19)	−0.008 (2)	0.010 (2)
C8	0.0255 (18)	0.035 (2)	0.035 (2)	−0.0030 (16)	0.0037 (16)	0.0117 (17)
C9	0.046 (2)	0.032 (2)	0.076 (3)	−0.0016 (19)	0.013 (2)	0.010 (2)
C10	0.0274 (19)	0.0260 (18)	0.038 (2)	−0.0042 (15)	0.0007 (16)	0.0009 (15)
C11	0.054 (3)	0.050 (2)	0.041 (2)	−0.005 (2)	−0.008 (2)	0.0116 (19)
C12	0.040 (2)	0.0230 (17)	0.034 (2)	−0.0067 (16)	−0.0057 (17)	0.0055 (16)
O16	0.120 (3)	0.070 (2)	0.0502 (19)	−0.002 (2)	0.002 (2)	0.0049 (17)
N1	0.063 (2)	0.045 (2)	0.053 (2)	−0.0038 (19)	0.0088 (19)	−0.0055 (18)
C13	0.063 (3)	0.066 (3)	0.058 (3)	0.007 (3)	−0.001 (2)	0.006 (2)
C14	0.039 (2)	0.042 (2)	0.061 (3)	0.0000 (19)	0.005 (2)	0.001 (2)

### Geometric parameters (Å, °)

**Table:**

Fe1—O13	1.897 (2)	O12—C12	1.272 (4)
Fe1—O1	1.987 (2)	O17—N1	1.260 (4)
Fe1—O10	1.995 (2)	O18—N1	1.240 (4)
Fe1—O12	2.005 (3)	O14—C14	1.316 (4)
Fe1—O3	2.063 (2)	O14—H14	0.8200
Fe1—O1W	2.104 (2)	O15—C14	1.209 (5)
Fe2—O13	1.900 (2)	C1—C2	1.494 (5)
Fe2—O5	1.985 (2)	C1—H1A	0.9600
Fe2—O7	2.021 (2)	C1—H1B	0.9600
Fe2—O2	2.030 (2)	C1—H1C	0.9600
Fe2—O4	2.030 (2)	C3—C4	1.501 (5)
Fe2—O2W	2.126 (3)	C3—H3A	0.9600
Fe3—O13	1.916 (2)	C3—H3B	0.9600
Fe3—O11	2.011 (3)	C3—H3C	0.9600
Fe3—O6	2.013 (2)	C5—C6	1.500 (5)
Fe3—O8	2.013 (2)	C5—H5A	0.9600
Fe3—O9	2.017 (2)	C5—H5B	0.9600
Fe3—O3W	2.048 (2)	C5—H5C	0.9600
O1W—H1AW	0.815 (9)	C7—C8	1.497 (5)
O1W—H1BW	0.814 (9)	C7—H7A	0.9600
O2W—H2AW	0.816 (9)	C7—H7B	0.9600
O2W—H2BW	0.819 (9)	C7—H7C	0.9600
O3W—H3AW	0.816 (9)	C9—C10	1.497 (5)
O3W—H3BW	0.818 (9)	C9—H9A	0.9600
O1—C2	1.260 (4)	C9—H9B	0.9600
O2—C2	1.251 (4)	C9—H9C	0.9600
O3—C4	1.258 (4)	C11—C12	1.499 (5)
O4—C4	1.260 (4)	C11—H11A	0.9600
O5—C6	1.249 (4)	C11—H11B	0.9600
O6—C6	1.270 (4)	C11—H11C	0.9600
O7—C8	1.254 (4)	O16—N1	1.229 (5)
O8—C8	1.268 (4)	C13—C14	1.475 (6)
O9—C10	1.251 (4)	C13—H13A	0.9600
O10—C10	1.251 (4)	C13—H13B	0.9600
O11—C12	1.241 (4)	C13—H13C	0.9600
			
O13—Fe1—O1	95.13 (10)	Fe2—O13—Fe3	119.60 (11)
O13—Fe1—O10	98.80 (9)	C14—O14—H14	109.5
O1—Fe1—O10	165.92 (10)	C2—C1—H1A	109.5
O13—Fe1—O12	94.40 (10)	C2—C1—H1B	109.5
O1—Fe1—O12	88.79 (12)	H1A—C1—H1B	109.5
O10—Fe1—O12	92.13 (11)	C2—C1—H1C	109.5
O13—Fe1—O3	92.81 (9)	H1A—C1—H1C	109.5
O1—Fe1—O3	92.29 (11)	H1B—C1—H1C	109.5
O10—Fe1—O3	85.04 (11)	O2—C2—O1	123.8 (3)
O12—Fe1—O3	172.58 (10)	O2—C2—C1	118.9 (3)
O13—Fe1—O1W	176.56 (9)	O1—C2—C1	117.4 (3)
O1—Fe1—O1W	81.82 (10)	C4—C3—H3A	109.5
O10—Fe1—O1W	84.19 (9)	C4—C3—H3B	109.5
O12—Fe1—O1W	87.14 (10)	H3A—C3—H3B	109.5
O3—Fe1—O1W	85.75 (10)	C4—C3—H3C	109.5
O13—Fe2—O5	97.45 (9)	H3A—C3—H3C	109.5
O13—Fe2—O7	94.56 (9)	H3B—C3—H3C	109.5
O5—Fe2—O7	90.68 (10)	O3—C4—O4	124.8 (3)
O13—Fe2—O2	94.67 (9)	O3—C4—C3	118.2 (3)
O5—Fe2—O2	87.64 (10)	O4—C4—C3	117.0 (3)
O7—Fe2—O2	170.76 (10)	C6—C5—H5A	109.5
O13—Fe2—O4	94.54 (9)	C6—C5—H5B	109.5
O5—Fe2—O4	167.95 (10)	H5A—C5—H5B	109.5
O7—Fe2—O4	89.59 (10)	C6—C5—H5C	109.5
O2—Fe2—O4	90.17 (10)	H5A—C5—H5C	109.5
O13—Fe2—O2W	174.84 (10)	H5B—C5—H5C	109.5
O5—Fe2—O2W	87.69 (11)	O5—C6—O6	124.6 (3)
O7—Fe2—O2W	85.90 (10)	O5—C6—C5	118.9 (3)
O2—Fe2—O2W	84.95 (10)	O6—C6—C5	116.5 (3)
O4—Fe2—O2W	80.32 (10)	C8—C7—H7A	109.5
O13—Fe3—O11	92.62 (10)	C8—C7—H7B	109.5
O13—Fe3—O6	96.76 (9)	H7A—C7—H7B	109.5
O11—Fe3—O6	86.74 (11)	C8—C7—H7C	109.5
O13—Fe3—O8	93.76 (10)	H7A—C7—H7C	109.5
O11—Fe3—O8	173.00 (10)	H7B—C7—H7C	109.5
O6—Fe3—O8	89.64 (11)	O7—C8—O8	124.4 (3)
O13—Fe3—O9	94.77 (9)	O7—C8—C7	118.5 (3)
O11—Fe3—O9	91.68 (11)	O8—C8—C7	117.1 (3)
O6—Fe3—O9	168.42 (10)	C10—C9—H9A	109.5
O8—Fe3—O9	90.67 (10)	C10—C9—H9B	109.5
O13—Fe3—O3W	177.83 (10)	H9A—C9—H9B	109.5
O11—Fe3—O3W	86.91 (10)	C10—C9—H9C	109.5
O6—Fe3—O3W	85.33 (10)	H9A—C9—H9C	109.5
O8—Fe3—O3W	86.82 (10)	H9B—C9—H9C	109.5
O9—Fe3—O3W	83.13 (10)	O9—C10—O10	124.3 (3)
Fe1—O1W—H1AW	115.2 (13)	O9—C10—C9	118.1 (3)
Fe1—O1W—H1BW	116 (2)	O10—C10—C9	117.6 (3)
H1AW—O1W—H1BW	111.0 (16)	C12—C11—H11A	109.5
Fe2—O2W—H2AW	126.2 (19)	C12—C11—H11B	109.5
Fe2—O2W—H2BW	112 (3)	H11A—C11—H11B	109.5
H2AW—O2W—H2BW	109.8 (15)	C12—C11—H11C	109.5
Fe3—O3W—H3AW	126.8 (15)	H11A—C11—H11C	109.5
Fe3—O3W—H3BW	117.5 (16)	H11B—C11—H11C	109.5
H3AW—O3W—H3BW	110.7 (16)	O11—C12—O12	125.1 (3)
C2—O1—Fe1	135.3 (2)	O11—C12—C11	117.6 (3)
C2—O2—Fe2	129.6 (2)	O12—C12—C11	117.3 (3)
C4—O3—Fe1	127.6 (2)	O16—N1—O18	122.8 (4)
C4—O4—Fe2	134.9 (2)	O16—N1—O17	119.9 (4)
C6—O5—Fe2	134.0 (2)	O18—N1—O17	117.2 (4)
C6—O6—Fe3	132.3 (2)	C14—C13—H13A	109.5
C8—O7—Fe2	132.1 (2)	C14—C13—H13B	109.5
C8—O8—Fe3	134.2 (2)	H13A—C13—H13B	109.5
C10—O9—Fe3	135.5 (2)	C14—C13—H13C	109.5
C10—O10—Fe1	131.6 (2)	H13A—C13—H13C	109.5
C12—O11—Fe3	134.2 (2)	H13B—C13—H13C	109.5
C12—O12—Fe1	130.9 (2)	O15—C14—O14	121.4 (4)
Fe1—O13—Fe2	120.78 (11)	O15—C14—C13	124.1 (4)
Fe1—O13—Fe3	119.60 (11)	O14—C14—C13	114.5 (4)

### Hydrogen-bond geometry (Å, °)

**Table:**

*D*—H···*A*	*D*—H	H···*A*	*D*···*A*	*D*—H···*A*
O14—H14···O17^i^	0.82	1.82	2.642 (4)	178
O3W—H3AW···O15^ii^	0.816 (9)	1.894 (9)	2.697 (4)	168 (2)
O1W—H1AW···O18^iii^	0.815 (9)	2.008 (10)	2.821 (4)	176 (2)
O3W—H3BW···O17^iv^	0.818 (9)	1.938 (13)	2.742 (4)	167 (3)
O2W—H2AW···O15^v^	0.816 (9)	2.28 (2)	2.904 (4)	134 (2)
O1W—H1BW···O3^vi^	0.814 (9)	2.188 (12)	2.948 (3)	155 (2)

Symmetry codes: (i) −*x*+1, −*y*+1, −*z*+2; (ii) *x*−1, *y*, *z*; (iii) −*x*+1, *y*+1/2, −*z*+3/2; (iv) *x*, *y*+1, *z*; (v) *x*−1, −*y*+3/2, *z*−1/2; (vi) −*x*+1, −*y*+2, −*z*+1.
